# Dense Silicon Nanowire Networks Grown on a Stainless‐Steel Fiber Cloth: A Flexible and Robust Anode for Lithium‐Ion Batteries

**DOI:** 10.1002/adma.202105917

**Published:** 2021-10-22

**Authors:** Sumair Imtiaz, Ibrahim Saana Amiinu, Dylan Storan, Nilotpal Kapuria, Hugh Geaney, Tadhg Kennedy, Kevin M. Ryan

**Affiliations:** ^1^ Bernal Institute University of Limerick Limerick V94 T9PX Ireland; ^2^ Department of Chemical Sciences University of Limerick Limerick V94 T9PX Ireland; ^3^ Centre for Marine and Renewable Energy Ireland (MaREI) University of Limerick Limerick V94 T9PX Ireland

**Keywords:** fire‐resistant anodes, flexible electrodes, high mass loading, silicon nanowires, stainless‐steel fiber cloths

## Abstract

Silicon nanowires (Si NWs) are a promising anode material for lithium‐ion batteries (LIBs) due to their high specific capacity. Achieving adequate mass loadings for binder‐free Si NWs is restricted by low surface area, mechanically unstable and poorly conductive current collectors (CCs), as well as complicated/expensive fabrication routes. Herein, a tunable mass loading and dense Si NW growth on a conductive, flexible, fire‐resistant, and mechanically robust interwoven stainless‐steel fiber cloth (SSFC) using a simple glassware setup is reported. The SSFC CC facilitates dense growth of Si NWs where its open structure allows a buffer space for expansion/contraction during Li‐cycling. The Si NWs@SSFC anode displays a stable performance for 500 cycles with an average Coulombic efficiency of >99.5%. Galvanostatic cycling of the Si NWs@SSFC anode with a mass loading of 1.32 mg cm^−2^ achieves a stable areal capacity of ≈2 mAh cm^−2^ at 0.2 C after 200 cycles. Si NWs@SSFC anodes with different mass loadings are characterized before and after cycling by scanning and transmission electron microscopy to examine the effects of Li‐cycling on the morphology. Notably, this approach allows the large‐scale fabrication of robust and flexible binder‐free Si NWs@SSFC architectures, making it viable for practical applications in high energy density LIBs.

## Graphical abstract

A dense and tunable mass loading of Si NWs is directly grown on a highly conductive, flexible, fire‐resistant, and mechanically robust interwoven stainless‐steel fiber cloth (SSFC) by a simple and readily scalable glassware setup. Si NWs@SSFC anode with a mass loadings of 1.32 mg cm^−2^ achieves a stable areal capacity of ≈2 mAh cm^−2^ after 200 cycles.

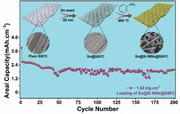

## 1 Introduction

Developing lithium‐ion batteries (LIBs) with higher capacity, longer cycle life, competitive cost, and higher energy density is essential to meet the demands of modern energy storage applications such as portable electronic devices and electric vehicles.^[^ ^1^ ^]^ As the energy density of current state‐of‐the‐art graphite intercalation anodes has reached its theoretical limit, a higher capacity anode material is vital to further increase the energy density of LIBs. Silicon (Si) has emerged as the most promising anode material for next generation LIBs as it offers a tenfold theoretical increase in lithium storage capacity over graphite (3579 vs 372 mAh g^−1^).^[^ ^2^ ^]^ Compared to other high‐capacity materials (i.e., Ge, Sn, and In), Si also exhibits multiple advantages such as huge natural abundance, relative low‐cost, environmental friendliness and low working voltage(≈0.4 V vs Li^+^/Li).^[^ ^3^ ^]^ However, Si undergoes significant volume change (≈300%) during lithiation and delithiation, resulting severe cracking, pulverization, and loss of contact of the active material with the CCs.^[^ ^4^ ^]^

From a material design viewpoint, a plausible way to address the above challenges is to reduce the size of Si anode down to the nanoscale. Pioneering studies have found that below a critical size of ≈150 nm, cracking and pulverization of Si is limited^[^ ^5^ ^]^ due to the high surface‐to‐volume ratio and enhanced stress resistance. Several strategies have been investigated on morphology tuning that results in improved performances, including Si nanoparticles,^[^ ^6^ ^]^ nanospheres,^[^ ^7^ ^]^ nanotubes,^[^ ^8^ ^]^ nanowires (NWs),^[^ ^9^ ^]^ and thin nanofilms.^[^ ^10^ ^]^ Among different nanostructures, Si NWs have been considered as the most attractive candidate because the 1D structures can alleviate stress and accommodate large strains as they can expand both radially and longitudinally.^[^ ^11^ ^]^ NWs also provide direct current paths to facilitate good electrical conductivity, a relatively high surface area for good electrode‐electrolyte contact, improved electronic transport, and shorter ion diffusion distance.^[^ ^12^ ^]^ In addition, NWs can be directly grown on the CC, thus, eliminating the requirement for inactive electrode additives and binders, which is essential for improving the energy density compared to conventional slurry‐based electrodes.^[^ ^11^, ^13^ ^]^

One main challenge associated with the real‐world prospects of directly grown Si NWs is the low areal mass loadings.^[^ ^11^, ^14^ ^]^ Most of the directly grown Si NWs reported in the literature are based on rigid planar metal substrates with relatively low mass loadings, typically in the range of 0.18–0.40 mg cm^−2^.^[^ ^13^, ^15^ ^]^ As a result, the areal capacity of Si NW anodes is typically low (≤1 mAh cm^−2^). Metal foams such as Cu and Ni have shown improvement in terms of facilitating higher loadings owing to their relatively high surface area.^[^ ^15^, ^16^ ^]^ However, these substrates are not shown to be sufficiently mechanically robust for practical batteries. Carbon fiber cloth (CFC) has been employed to obtain dense growth of Si NWs using chemical vapor deposition,^[^ ^17^ ^]^ although the low electrical conductivity of CFC compared to metallic foils, and use of inactive metal seed catalyst (e.g., Ni,^[^ ^17c^ ^]^ Au^[^ ^17b^ ^]^) mitigates against optimal electrochemical performances. In addition, the high equipment cost and the difficulties for mass production of high quality binder‐free Si NWs have rendered them unattractive.^[^ ^18^ ^]^ Achieving a highly effective and scalable fabrication route for the high mass loading and dense growth of Si NWs on a mechanical robust and highly conductive substrate is desirable for practical LIBs.

Here, we have utilized a highly conductive, flexible, flame‐retardant and mechanically robust interwoven stainless‐steel fiber cloth (SSFC) for the growth of dense Sn‐seeded Si NW networks in a simple glassware setup. The SSFC is highly advantageous as the open structure of the SSFC permits buffer space for the Si NWs during cycling and promotes fast electronic/ionic transport. The SSFC (individual fiber diameter: 8 µm) allows for the dense growth and uniform distribution of Sn‐seeded Si NWs with areal loadings in the range of 0.24–1.32 mg cm^−2^. The Si NWs@SSFC with the highest mass loading (1.32 mg cm^−2^) achieved an areal charge capacity of 4.3 and 3.9 mAh cm^−2^ at C/10 and C/5, respectively. The Si NWs@SSFC anodes further display good cycling stability for over 500 cycles attaining an average Coulombic efficiency greater than 99.5%. More importantly, the Si NWs@SSFC anode can be readily scaled up without compromising its structural integrity, which is highly desirable for practical batteries.

## 2 Results and Discussion

**Figure 1:**
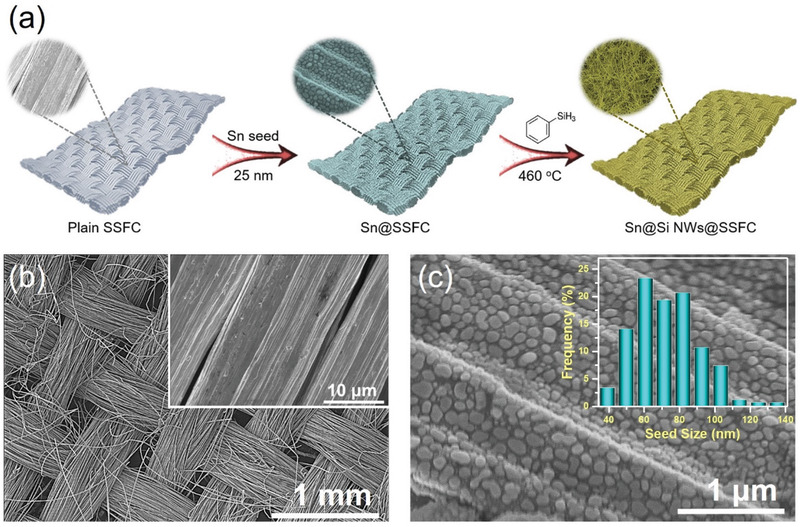
a) Schematic illustration of the synthesis of the flexible and dense growth of Sn‐seeded Si NWs@SSFC and corresponding magnified SEM images showing the plain SSFC, Sn‐seed coated SSFC and Sn‐seeded Si NWs@SSFC; b) low‐magnification SEM image of pristine SSFC, an inset showing the SEM image of individual fibers; and c) high‐magnification SEM images of Sn‐seed coated SSFC, an inset representing average Sn‐seed size distribution.

The synthesis of the flexible Si NWs@SSFC and corresponding scanning electron microscopy (SEM) images is schematically presented in **Figure** **1**a. The Low‐magnification SEM image of the pristine SSFC in Figure 1b clearly indicates an interwoven structure of the cloth, where an inset shows the individual fibers with a diameter of ≈8 µm. The SEM images of Sn‐seed coated SSFC in Figure 1c shows uniform coverage of individual fibers with well‐formed and isolated small‐sized Sn‐seeds with the average seed‐size ≈72 nm (inset Figure 1c). This dense and even distribution of Sn catalyst seeds on such an interwoven SSFC can allow for high density Si NW growth and high mass loading.^[^ ^19^ ^]^ The areal loading of Si NWs@SSFC was controlled by using either a one or dual sided Sn‐seed coated SSFC, reaction time (10–30 min.) and an amount of PS from 0.7–1.4 mL, as presented in Figure S1 (Supporting Information). Four different samples of Si NWs@SSFC with an average mass loadings of 0.24, 0.52, 1.03, and 1.32 mg cm^−2^ were obtained by varying these parameters. There was a visual change of color of the SSFC, which turned from light yellow/brown to brown with increasing mass loadings of Si NWs, as shown in Figure S2 (Supporting Information).

**Figure 2:**
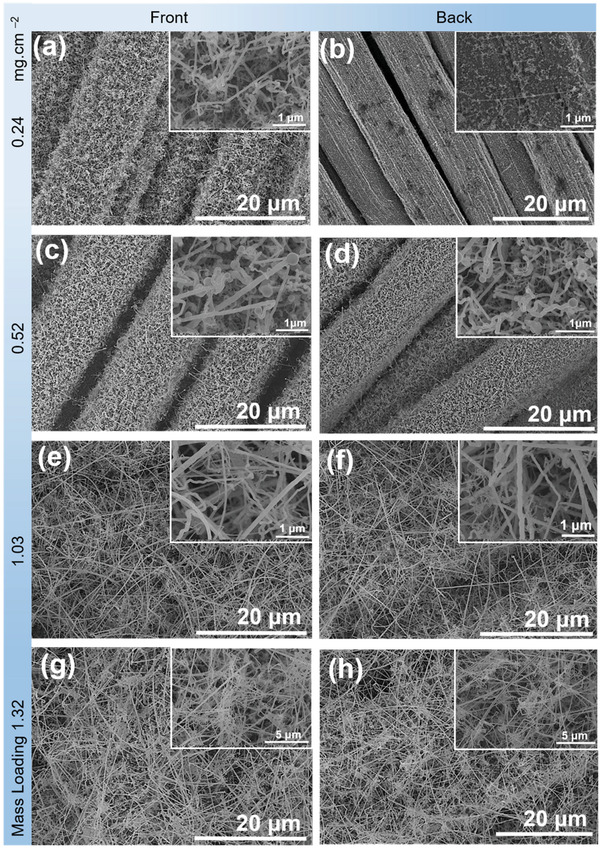
SEM images of the Si NWs@SSFC anodes at different magnifications from the front and back sides for a,b) 0.24 mg cm^−2^, c,d) 0.52 mg cm^−2^, e,f) 1.03 mg cm^−2^, and g,h) 1.32 mg cm^−2^ mass loading, respectively.

The SEM images in **Figure** **2**a–h show the front side (left panel) and back side (right panel) of the Si NWs@SSFC with different mass loading of 0.24, 0.52, 1.03 and 1.32 mg cm^−2^. All the samples clearly display dense growth and excellent coverage of Si NWs on both sides of SSFC except for the 0.24 mg cm^−2^ loading (Figure 2b), where the Sn seeds were deliberately coated on only the front side to obtain low loading of Si NWs. The individual SS fibers of the cloth can still be clearly observed in Figure 2a–d, whereas it is hard to distinguish for the higher loadings (Figure 2e–h). The spaces between the fibers were filled by the Si NWs for higher loadings (Figure 2e–h) and exhibit an intertwined network of Si NWs. This was enabled by dense growth of Si NWs through the fiber spaces, completely wrapping the SS fibers through intertwining leading to high and dense loading of the Si NWs@SSFC with an excellent surface coverage. Sporadic micrometer sized amorphous Si particles were also observed for the high loading sample 1.32 mg cm^−2^ (Figure S3a, Supporting Information), which are common by‐products of the synthesis of Si NWs with phenylsilane (PS).^[^ ^20^ ^]^ These particles add inactive mass to the substrate and could even lower the overall electrochemical performance.^[^ ^20^ ^]^ Therefore, they were removed from the Si NWs@SSFC samples by quick sonication for ≈30–60 s before calculating the mass of Si NWs. Furthermore, the 1.32 mg cm^−2^ sample showed more clustering of the Si NWs as clearly seen in Figure S3b,c (Supporting Information), further attesting to the high‐density growth features on the SSFC. The well‐ordered interwoven structure of the SSFC remains fully in tact after the reaction, demonstrating its robustness and suitability for use as a binder‐free CC (Figure S4, Supporting Information).

**Figure 3:**
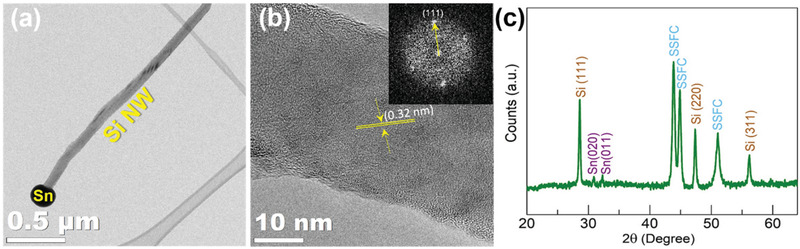
a) TEM image of a single Sn‐seeded Si NW and b) HRTEM image, and inset is the selected area FFT diffraction pattern, c) XRD pattern of Sn‐seeded Si NWs@SSFC.

**Figure** **3**a shows the representative transmission electron microscopy (TEM) image of a single Sn‐seeded Si NW. High resolution TEM (HRTEM) image (Figure 3b) of the Si NWs reveals lattice features typical of a crystalline Si,^[^ ^9^, ^21^ ^]^ exhibiting an FFT pattern indexed to the (111) planes and a lattice spacing of 0.32 nm, which is consistent with the XRD data (Figure 3c). Figure S5a–c (Supporting Information) shows the HRTEM images for the 0.52, 1.30, and 1.32 samples with the growth time of 10, 15, and 30 min, respectively. As the reaction time increases, a thin amorphous layer develops on the surface of the Si NWs. The average thickness of the amorphous shell is about 2, 2.5, and 6.5 nm for the 0.52, 1.30, and 1.32 mg cm^−2^ samples, respectively, and is consistent with previous studies, where an amorphous shell was observed with longer reaction time.^[^ ^17^, ^22^ ^]^

**Figure 4:**
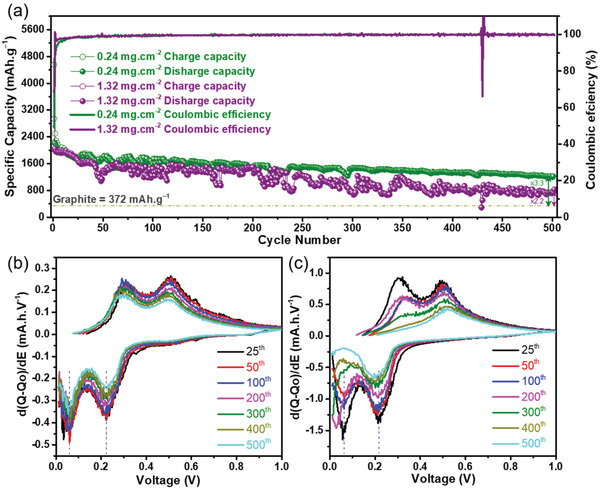
Electrochemical performance of the Sn‐seeded Si NWs@SSFC anode. a) Long‐term cycling stability of 0.24 and 1.32 mg cm^−2^ mass loading samples, Differential capacity plots of 25th, 50th, 100th, 200th, 300th, 400th, and 500th cycles for b) 0.24 and (c) 1.32 mg cm^−2^ sample.

The electrochemical performance of Si NWs@SSFC as a Li‐ion anode material was assessed in a half‐cell configuration in a Swagelok type cell. An experimental specific capacity is used to calculate the C‐rates, which is determined based on the constant current (350 mA g^−1^) charge–discharge of Sn‐seeded Si NWs@SSFC (Figure S6, Supporting Information). A C/5 rate represents a 5 h charge/discharge based on the experimental specific capacity of 2200 mAh g^−1^ for Sn‐seeded Si NW@SSFC.^[^ ^8^, ^23^ ^]^ The cycling performance of all electrodes at C/5 is presented in Figure S7 (Supporting Information). The initial reversible capacities are 2233.5, 2185.1, 2029, and 2019.2 for electrodes with 0.24, 0.52, 1.03, and 1.32 mg cm^−2^ mass loadings, respectively, and corresponding capacities of 1735.8, 1486.7, 1290.8, and 1241.5 mAh g^−1^ were retained after 110 cycles. The differential capacity plot (DCP) of 0.24 mg cm^−2^ sample indicates that both Si and Sn components contribute to the anodes capacity (Figure S8a, Supporting Information). The DCP of 0.24 mg cm^−2^ sample shows a major lithiation peaks at ≈50 mV and below ≈100 mV and delithiation peak at ≈300 mV and pronounced peak at ≈0.45 mV, which correspond to the characteristic peaks of the phase transitions of Si.^[^ ^9^, ^15^, ^24^ ^]^ Reversible cycling of Sn is also evident as the sharp lithiation peak at ≈400 mV and delithiation peaks at ≈600, ≈700, and ≈780 mV corresponds to the peaks observed when only Sn@SSFC are cycled. Notably, no apparent peak of Sn were observed for the high loading samples 0.52, 1.03, and 1.32 mg cm^−2^, which is most likely due to the signals being overshadowed by the high peak current originating from highly loaded Si^[^ ^21^, ^25^ ^]^ (Figure S8b–d, Supporting Information). However, based on the amount of Sn (0.047 mg cm^−2^ for 25 nm single layer, and ≈0.097 mg cm^−2^ for 25/25 nm dual layer on SSFC), the maximum attainable capacity from Sn at C/5 for 0.24, 0.52, 1.03 and 1.32 mg cm^−2^ Sn seeded Si NWs samples is 104.97, 211.95, 196.81, 195.86 mAh g^−1^, respectively. A prolonged cycling of the 0.24 and 1.32 mg cm^−2^ electrodes is presented in **Figure** **4**a. The Si NWs@SSFC with 0.24 mg cm^−2^ mass loading maintains an outstanding cycling stability, delivering a capacity of 1221.1 mAh g^−1^ after 500 cycles, whereas the high mass loading of 1.32 mg cm^−2^ delivered a capacity of 826.3 mAh g^−1^ that is 2.2 times the capacity of graphite. The initial charge/discharge voltage profiles for four different loadings (0.24, 0.52, 1.03, and 1.32 mg cm^−2^) at C/5 are shown in Figure S9a (Supporting Information), which indicates that the lithiation and delithiation of Si NWs@SSFC occur at similar potentials. As shown in Figure S9b (Supporting Information), the initial Coulombic efficiency (ICE) value increased with increasing mass loading of the Si NWs. This phenomenon is most likely related to the formation of SEI layer, as in the low mass loadings a higher surface area is in contact with the electrolyte, whereas in the high mass loadings, the contact area is relatively lower.^[^ ^26^ ^]^ As a result, the ICE increases from 49.04% to 68.63% when the Si NW mass loadings was increased from 0.24 to 1.32 mg cm^−2^, respectively. A similar phenomenon was also reported previously,^[^ ^26^ ^]^ where higher loadings exhibited higher ICE, primarily owing to the less exposure of the surface area to the electrolyte due to a higher density of NWs.

The comparison of electrochemical impedance spectroscopy (EIS) of all four electrodes after 1st and 10th cycle is presented in Figure S10 (Supporting Information). The equivalent circuit used in the analysis is shown in the inset of Figure S10a (Supporting Information). Where, *R*_s_ represents the electrolyte resistance, *R*_sur_ is the resistance at the surface of Si NWs and is attributed to the combination of charge transfer between the SEI and the Si NWs, Q1 is the constant phase elements and W is Warburg impedance related to Li diffusion.^[^ ^2^, ^27^ ^]^ The *R*_sur_ values for different loadings are listed in Table S1 (Supporting Information). After the 1st cycle, *R*_sur_ values are higher for the lower loading anode. This is apparent by the larger diameter of the semicircles signifies higher resistances in the high frequency region which is probably due to the greater resistance of the SEI owing to the larger area in contact to the electrolyte. Indeed, the diameter of the semicircle and *R*_sur_ value for 0.24, 0.52, and 1.03 mg cm^−2^ decreases after 10th cycle, signifying stable SEI formation and charge transfer properties with good internal connectivity and mechanical integrity of Si NWs@SSFC. However, an increase in the diameter of semicircle and *R*_sur_ occurs for 1.32 mg cm^−2^ electrode, which may be attributed to the relatively thicker SEI layer build up on the densely packed Si NWs@SSFC.^[^ ^28^ ^]^ Differential capacity analysis of the prolonged cycling of 0.24 and 1.32 mg cm^−2^ for the 25th, 50th, 100th, 200th, 300th, 400th, and 500th cycles are shown in Figure 4b,c, respectively. The DCP illustrates that the peak locations for lithiation/delithiation of 0.24 mg cm^−2^ sample remain constant even after 500 cycles (Figure 4b). The DCP of 1.32 mg cm^−2^ sample also showed similar characteristic peaks of Si NWs during lithiation and delithiation. Upon close examination, it can be seen that the lithiation peaks for 1.32 mg cm^−2^ sample shift to lower potential after 200 cycles onward and diminished for the following cycles (Figure 4c). It is observed that after this point, the delithiation peak at ≈0.3 mV associated to the lithiation peak of ≈65 mV started to decrease in intensity and after 300 cycles, the plateaus become a single plateau for the lithiation/delithiation process. This indicates that overpotential grows for the high mass loading sample after 200 cycles that shifts the reaction potentials due to polarization and the 1.32 mg cm^−2^ cell reaches its lower cutoff voltage limitation of 10 mV before complete lithiation of the Si NWs can occur.^[^ ^28^, ^29^ ^]^ Therefore, capacity for high mass loading of 1.32 mg cm^−2^ cell fades due to the incomplete lithiation and inhibited Li^+^ diffusion.^[^ ^30^ ^]^

**Figure 5:**
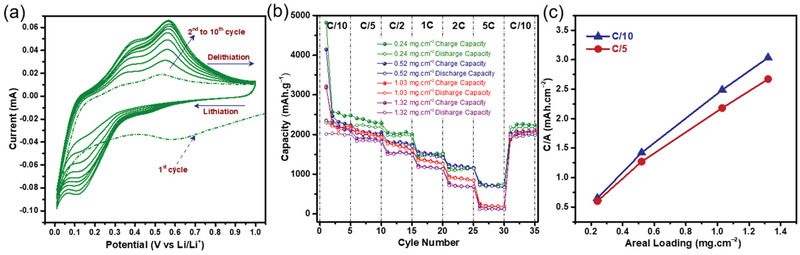
a) CV profiles of the 0.24 mg cm^−2^ sample. b) Rate capability of 0.24, 0.52, 1.03, and 1.32 mg cm^−2^ at various C‐rates, and c) areal capacity (C/A) versus areal loading at C/10 and C/5.

Cyclic voltammetry (CV) in **Figure** **5**a of the SiNWs@SSFC generates scan profiles of a typical Si lithiation/delithiation.^[^ ^9^, ^19^, ^31^ ^]^ During the first cycle, the cathodic peak around 600 mV is due to the formation of SEI layer and the peak at ≈150 mV corresponds to the lithiation of crystalline Si. At the first anodic scan, the peak at 540 mV is allocated to the delithiation process. In the following cathodic scans, the peaks at ≈150 mV and below ≈100 mV correspond to the lithiated conversion of amorphous and crystalline Si, respectively, while the anodic peaks corresponding to the delithiation of Si NWs appear at ≈390 and ≈560 mV, respectively. The cathodic and anodic peaks display a continuous enlargement of the current as the cycle number increases, demonstrating the incomplete lithiation of Si occurring in the first few cycles that gradually increases. Moreover, the CV curves tend to overlap after the first few cycles, signifying the high reversibility and stabilization of the lithiation/delithiation processes. Rate capability performance for each of the loadings were analyzed at C/10, C/5, C/2, C, 2 C, and 5 C (Figure 5b). The anodes with low mass loading exhibit the highest capacities at low C‐rates. It is apparent that anodes with low mass loadings of 0.24 and 0.52 mg cm^−2^ sustain the capacities with a slight depletion at higher C‐rates, retaining reversible capacities of 795 and 781 mAh g^−1^ at 5 C, respectively. The high mass loading anodes also exhibit excellent performance up to 2 C, with capacities of 254 and 184.6 mAh g^−1^ for the 1.03 and 1.32 mg cm^−2^ sample at 5 C. Nevertheless, all of the four different loading samples recover their capacities when the rate returned abruptly from 5 C to the initial rate of C/10.

Attaining a high areal capacity is essential to qualify the Si NWs@SSFC anodes for practical application. Therefore, the influence of mass loadings of the Si NWs@SSFC on the areal capacity was also evaluated. The Figure 5c shows a linear increase in the areal capacity with the mass loading of Si NWs, where the Si NWs@SSFC with 1.32 mg cm^−2^ loading yields an areal reversible capacity of 3.03 mAh cm^−2^ at C/10. After 50 cycles, the Si NWs@SSFC with the mass loadings of 0.24, 0.52, 1.03, and 1.32 mg cm^−2^ delivered areal capacities of 0.49, 1.06, 1.98, and 2.19 mAh cm^−2^, respectively (Figure S11, Supporting Information). Even after prolonged cycling, the 1.32 mg cm^−2^ sample achieves a stable areal capacity of ≈2 mAh cm^−2^ for over 200 cycles at C/5, which is 5.5 times higher than that of the 0.24 mg cm^−2^ sample (Figure S12, Supporting Information), further endorsing the ability of SSFC to obtain high mass loadings and high achievable areal capacity. It is worth noting that the SSFC alone did not show any capacity and remains inert during charge/discharge (Figure S13a, Supporting Information). However, the robust interconnected Si NWs@SSFC architecture yields a high reversible capacity of 2233.5 mAh g^−1^ compared to 1865.2 mAh g^−1^ for Si NWs@SS foil with comparable mass loadings at C/5 (Figure S13b, Supporting Information). Such a flexible metallic CC is beneficial for achieving high mechanical robustness, fast electron transfer and electrolyte diffusion without compromising the conductivity. In addition, the open‐structure offers a buffer space for Si NWs to expand freely during charge/discharge and improve battery electrochemical performance.

**Figure 6:**
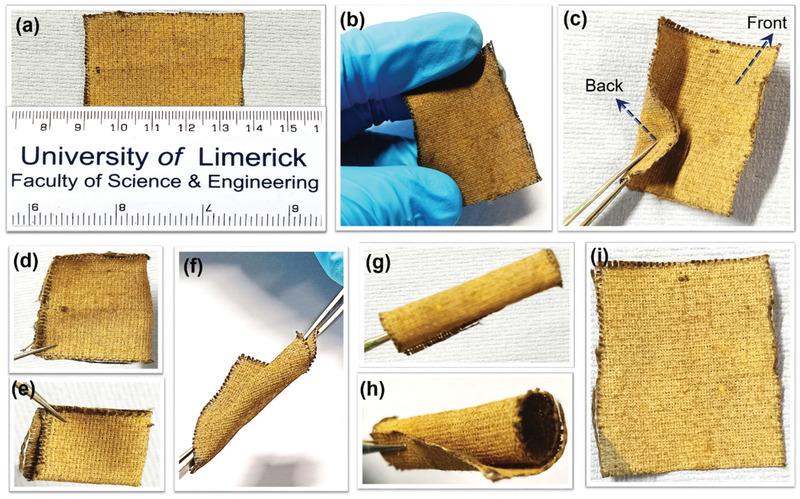
a–i) Digital photographs of the Sn‐seeded Si NWs@SSFC anode, illustrating mechanical robustness under various conditions of d) single folding, e) multiple folding, f) twisting, rolling g) top and h) side view, and i) after releasing state, showing no deformation.

It is worth noting that the Si NWs@SSFC exhibited high mechanical robustness and flexibility with the Si NWs sustaining intense multiple folding, twisting, and rolling without any damage or delamination as demonstrated in **Figure** **6**, Figure S14 (Supporting Information) and Video S1 (Supporting Information). The Si NWs@SSFC anode was cycled at C/5 after this intense mechanical handling, and still sustained a stable cycling performance (Figure S15, Supporting Information), which affirm the mechanical resilience of the Si NWs@SSFC anode. To further check the mechanical adherence of Si NWs with SSFC, a sonication test was performed on the Si NWs@SSFC and Si NWs@SS foil for 60 min in toluene. The Si NWs@SS shows a characteristic yellow/brown color dispersion of Si NWs in just 1 min of sonication (delamination), whereas for the Si NWs@SSFC solution remained colorless even after 60 min (Figure S16, Supporting Information), confirming the high mechanical stability of the anode. In addition, the weight loss of Si NWs@SS foil after 60 min of sonication was 84.15%, compared to only 7.52% for the Si NWs@SSFC, demonstrating the greatly enhanced adhesion of Si NWs with SSFC. A flammability test (Figure S17 and Video S2, Supporting Information) indicates that the Si NWs@SSFC anode is fire resistant when directly exposed to a flame compared to Si NWs grown on a flexible CFC (Si NWs@CFC) with similar interwoven features and have been largely employed as a flexible substrate in many reports.^[^ ^17^, ^18^ ^]^ The Si NWs@CFC instantly flamed for a period that is long enough to cause catastrophic fire damage. After exposure to the fire, the Si NWs@SSFC anode still retained its structural integrity of high flexibility and mechanical robustness, whereas the Si NWs@CFC lost all its mechanical integrity and could be easily crushed to powder. To further realistically mimic the effect of fire on the Si NWs@SSFC anode, we tested it in a half‐cell against Li after exposure to fire. As demonstrated in Figure S18 (Supporting Information) the anode displays a stable cycling efficiency without any degradation in performance. This mechanical and flame‐resistant nature of the Si NWs@SSFC is crucial and provides additional safety features to the battery during high temperatures operation and physical abuse. Consequently, the Si NWs@SSFC anode is not only advantageous for obtaining high energy density LIBs but also can lead to more reliable and safer batteries. Moreover, such a flexible and robust architecture can provide additional advantages, including higher conductivity, as compared to CFC and high‐density Si NWs growth without compromising structural integrity. The open structure of the SSFC promotes efficient electrolyte penetration through the whole structure of the anode while also providing a buffer zone to accommodate any severe volume expansion during cycling. Remarkably, the Si NWs@SSFC is readily scalable, shown here as substrates 26 cm long with an area of 130 cm^2^ achieved by this simple glassware‐based method (Figure S19, Supporting Information).

**Figure 7:**
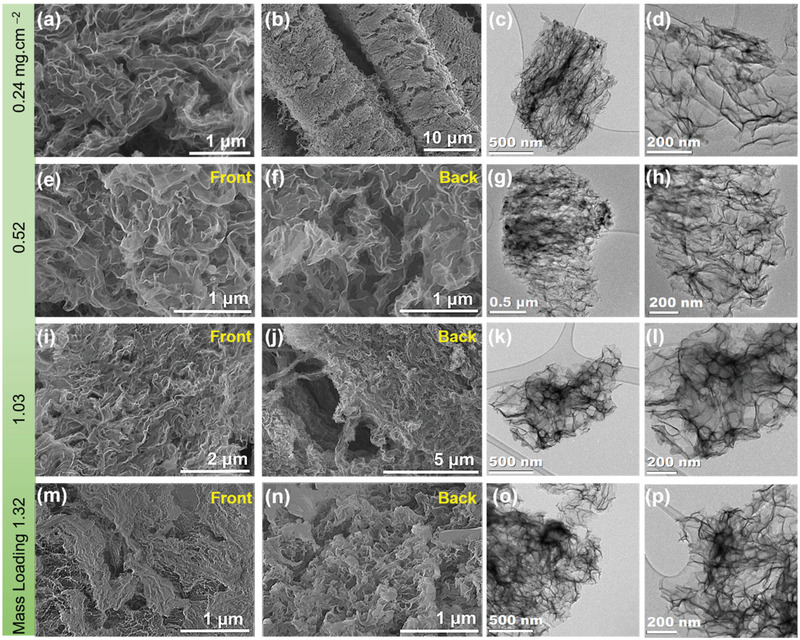
Ex situ SEM images of Si NWs@SSFC anodes after 50 cycles: a,b) 0.24 mg cm^−2^ (front sides), e) front and f) backside of 0.52 mg cm^−2^, i) front and j) backside of 1.03 mg cm^−2^, and m) front and n) backside of 1.32 mg cm^−2^ sample showing that the original Si NWs have transformed to a network of interconnected material. Low‐ and high‐magnification TEM images: c,d) 0.24 mg cm^−2^, g,h) 0.52 mg cm^−2^, k,l) 1.03 mg cm^−2^, and o,p) 1.32 mg cm^−2^ sample. The complete restructuring of Si NWs to form a porous network of interconnected ligaments due to the cumulative effect of pore formation and Li assisted welding of Si NWs is apparent.

We have also evaluated the effect of cycling on the morphology of all four different anodes post‐cycling via ex situ electron microscopic study. Figure S20 (Supporting Information) shows digital photos of the anodes after cycling. It is noticeable that the no physical surface crack or damage is observed which indicates that Si NWs@SSFC anodes can maintain mechanical strength during battery operations. **Figure** **7** presents the ex situ SEM and TEM images of all four different samples after 50 cycles. The ex situ SEM and TEM images reveal that despite the highly dense loadings, all samples significantly transform into a porous network of interconnected ligaments in agreement with our previous studies.^[^ ^11^, ^13^, ^25^ ^]^ This porous network is highly advantageous as it is mechanically robust and once formed exhibits no significant deformation upon further charge/discharge. The available spaces in the porous network can provide the additional buffer zone to accommodate the volume variations during lithiation/delithiation.^[^ ^21^ ^]^ The benefits of SSFC is evident from the stable performance obtained by Si NWs@SSFC anode even at high mass loadings. As in a recent study, this ideal restructuring was not observed on SS foil when the loading of NWs was increased beyond 0.6 mg cm^−2^.^[^ ^26^ ^]^ The low‐magnification ex situ SEM images in Figure S21a–c (Supporting Information) illustrates that the porous network ligaments remain well adhered to the SSFC substrate for 0.24, 0.52, and 1.03 mg samples. A slight delamination and partly uncycled Si NWs occur at some instances for the 1.32 mg sample (Figure S21d–f, Supporting Information). This directly reflects the cycling behavior of 1.32 mg, where a drop in capacity and an attenuation of the peak was observed after 50 cycles (Figure 4a,c). However, both the capacity (Figure 4a) and peak intensity (Figure 4c) recovered afterward. This indicates that the Si NWs remained in good contact with SSFC due to the intertwined network.

Overall, the scalable and simple synthesis protocol of Si NWs for direct growth on fire‐resistant, conductive, flexible, and mechanically robust SSFC and electrochemical performance of the obtained Si NWs@SSFC at high loadings offers advantages over most previously published studies involving binder‐free, composites and conventional slurry‐based Si anodes as presented in Table S2 (Supporting Information). This demonstrates the high promise of the Si NWs@SSFC architectures for practical applications in high‐energy density LIBs.

## 3 Conclusion

In summary, we demonstrate an efficient approach to achieve tunable mass loading of Si NWs directly grown on a highly flexible, conductive, flame‐retardant and mechanically robust SSFC CC in a simple glassware setup. The interwoven features of the SSFC CC enabled high density loading of the catalyst seed, leading to a high Si NW loading in excess of 1.3 mg cm^−2^. The Si NWs@SSFC anodes with areal loading of 0.24 and 1.32 mg cm^−2^ display a stable cycling performance for over 500 cycles with the average Coulombic efficiencies exceeding 99.5%. The anode with a mass loading of 1.32 mg cm^−2^ achieved a stable areal capacity of ≈2 mAh cm^−2^ at C/5 even after 200 cycles. The ex situ SEM and TEM analysis of the Si NWs@SSFC anodes after extended cycling show a complete restructuring of the Si NWs into a highly stable porous network structure owing to the cumulative effect of pore formation and Li assisted welding. Significantly, the binder‐free Si NWs@SSFC anode is also fire‐resistant and scalable in large samples without compromising mechanical integrity or electrochemical capability, which are key practical anode requirements for the next generation of LIBs for portable electronic devices and electric vehicles.

## 4 Experimental Section

### Substrate Preparation

SSFC (weight: 38 mg cm^2^ and thickness: ≈0.35 mm) was purchased from 3L Tex., Co. Ltd. A piece of the SSFC was ultrasonically washed in acetone and deionized (DI) water, followed by drying at 70 °C in an oven. After that, a 25 nm layer of Sn (99.999%, Kurt J. Lesker) as catalyst seeds was thermally evaporated on to the surface of the SSFC in a glove box‐based evaporation unit (Mbraun, MB‐200B). The Sn seeds were deposited on either one or both sides of the SSFC depending on the required amount of Si NWs loading, providing to the average mass loading of 0.047 and 0.097 mg cm^−2^ of Sn for 25 nm single side and 25/25 nm double sided SSFC, respectively. The Sn‐seed coated SSFC was stored in the Argon (Ar)‐filled glove box prior to reaction.

### Synthesis of Si Nanowires

Si NWs synthesis were carried out in a long‐neck round bottomed flask. A piece of the Sn‐seed coated SSFC substrate was slotted in a custom‐built steel holder and placed vertically around the neck area of the flask's bulb. The flask was then attached to a Schlenk line setup via a water condenser, and positioned inside a three‐zone type furnace. The temperature of the system was ramped up to 160 °C and a vacuum of ≈200 mTorr was applied for 30 minutes to remove any moisture. Subsequently, the system was purged with Ar gas, and the temperature was raised to 460 °C under a constant flow of Ar. A water condenser was employed to control the reflux and to keep the reaction under control. PS (97.0%, fluorochem) was injected into the system through a septum cap sealing the condenser. By varying the single or dual sided Sn‐seed coated SSFC, reaction time of 10–30 min, and 0.7–1.4 mL of PS, different mass loadings of Si NWs were achieved. Figure S1 (Supporting Information) provides the schematic of the reaction setup and summarizes the reaction conditions for four different reactions. The mass loading of the obtained Sn‐seeded Si NWs@SSFC samples ranges from 0.23–0.26 (condition #1), 0.50–0.54 (condition #2), 0.99–1.05 (condition #3), and 1.30–1.33 mg cm^−2^ (condition #4). For brevity, these samples are hereafter referred based on the average mass loadings as 0.24, 0.52, 1.03 and 1.32 mg cm^−2^, respectively. In all cases, the reaction was stopped by switching off the furnace and then allowed to cool naturally to room temperature. The Sn‐seeded Si NWs@SSFC sheets were extracted from the flask and washed with toluene, dried in air, cut into (0.7 × 0.7 cm) pieces and stored under Ar prior to cell assembly. As a control experiment, Sn seeds were also directly deposited on SS‐foil (0.1 mm thick, 99.999%, Kurt J. Lesker) and CFC (Kynol activated carbon fabric ACC‐507‐25) and then subjected to similar treatment for the growth of Si NWs. The large area substrate (5 × 26 cm) of Sn‐seeded Si NWs@SSFC was prepared in the same system with dual reactions (15 + 15 min) by inverting the sides of SSFC in the flask to obtain the conformal growth using ≈3.0 mL of PS.

### Materials Characterization

SEM analysis was carried out using a Hitachi SU‐70 system operating between 5 and 20 kV.  TEM analysis was conducted on JEOL JEM‐2100F field emission microscope equipped with a Gatan Ultrascan CCD camera and EDAX Genesis EDS detector and operated at 200 kV. For TEM analysis, the Si NWs were removed from the substrate through ultrasonication in toluene before being drop cast onto a lacey carbon TEM grid (200 mesh Cu). X‐ray diffraction patterns were collected on PANalytical Empyrean diffractometer equipped with a PIXcel^3D^ detector and CuKα radiation source (λ = 1.5406 Å), operating at 40 kV and 40 mA at room temperature. The masses of the Sn seeded Si NWs@SSFC were determined through careful measurement using a Sartorius Microbalance (Sartorius SE2, ± 0.25 µg repeatability). For post‐mortem analysis, SEI layer was removed by soaking the electrode in acetonitrile overnight, followed by rinsing in 0.1 × 10^−3^ m acetic acid, DI water and ethanol.

### Electrochemical Measurements

The electrochemical performance of the as synthesized Sn‐seeded Si NWs@SSFC anodes was evaluated using Swagelok type cells assembled in an Ar‐filled glove box. The cells consisted of the Sn‐seeded Si NWs@SSFC as the working electrode, Celgard membrane as a separator, and a lithium foil as the counter/reference electrode. A solution of 1 m LiPF_6_ in ethylene carbonate/diethyl carbonate (1:1 v/v) + 3% vinylene carbonate (VC) was used as the electrolyte. Galvanostatic measurements were carried out using a Biologic MPG‐2 in a potential range of 0.01–1.0 V (vs Li/Li^+^). The CV tests were performed at a scan rate of 0.1 mV.s^−1^, and EIS measurements were obtained by applying an AC voltage with an amplitude of 10 mV over the frequency range of 10 KHz to 10 mHz.

## Conflict of Interest

The authors declare no conflict of interest.

## Supporting information

Supporting Information

Supplemental Video 1

Supplemental Video 2

## Data Availability

The data that support the findings of this study are available from the corresponding author upon reasonable request.
