# Supplementary material for: Dense Silicon Nanowire Networks Grown on a Stainless‐Steel Fiber Cloth: A Flexible and Robust Anode for Lithium‐Ion Batteries
Source: Adv Mater. 2021 Oct 22;33(52):2105917. doi: 10.1002/adma.202105917 (PMC11469259; doi:10.1002/adma.202105917)
Supplement: Supplementary file 1 — Supporting Information [file ADMA-33-2105917-s001.pdf]

ADVANCED MATERIALS

Supporting Information

for *Adv. Mater.,* DOI: 10.1002/adma.202105917

Dense Silicon Nanowire Networks Grown on a Stainless-Steel Fiber Cloth: A Flexible and Robust Anode for Lithium-Ion Batteries

Sumair Imtiaz, Ibrahim Saana Amiinu, Dylan Storan, Nilotpal Kapuria, Hugh Geaney, Tadhg Kennedy, and Kevin M. Ryan**

Supporting Information

Dense Silicon Nanowire Networks Grown on a Stainless Steel Fiber Cloth: A Flexible and Robust Anode for Lithium-ion Batteries

Sumair Imtiaz, [a,b,c] *Ibrahim Saana Amiinu,*[a,b] *Dylan Storan,*[a,b] *Nilotpal Kapuria,* [a,b] *Hugh Geaney,* [a,b] *Tadhg Kennedy,* *[a,b] *and Kevin M. Ryan,**[a,b,c]

[a] Bernal Institute, University of Limerick, Limerick V94 T9PX, Ireland

[b] Department of Chemical Sciences, University of Limerick, Limerick V94 T9PX, Ireland

[c] Centre for Marine and Renewable Energy Ireland (MaREI), University of Limerick, Limerick V94 T9PX, Ireland

*Corresponding Author:

E-mail: tadhg.kennedy@ul.ie, Phone: +353-61 233732

E-mail: kevin.m.ryan@ul.ie, Phone: +353-61 213167

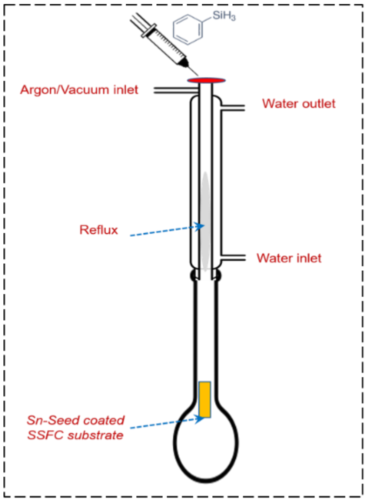

Condition #1<br>Loading ($mg.cm^{-2}$) = 0.23 – 0.26	
Sn Deposition	Single Side (25 nm)
Phenylsilane (mL)	0.70
Reaction Time (min.)	10

Condition #2<br>Loading ($mg.cm^{-2}$) = 0.50 – 0.54	
Sn Deposition	Dual Sided (25/25 nm)
Phenylsilane (mL)	0.70
Reaction Time (min.)	10

Condition #3<br>Loading ($mg.cm^{-2}$) = 0.99 – 1.05	
Sn Deposition	Dual Sided (25/25 nm)
Phenylsilane (mL)	0.70
Reaction Time (min.)	15

Condition #4<br>Loading ($mg.cm^{-2}$) = 1.30 – 1.33	
Sn Deposition	Dual Sided (25/25 nm)
Phenylsilane (mL)	0.70 + 0.70
Reaction Time (min.)	30

Figure S1. Reaction set up and conditions for obtaining the four different samples with different amount of loading ranges from 0.23 – 0.26 for reaction #1, 0.50 – 0.54 for reaction #2, 0.99 – 1.05 for reaction #3, and 1.30 – 1.33 $mg.cm^{-2}$ for reaction #4, respectively.

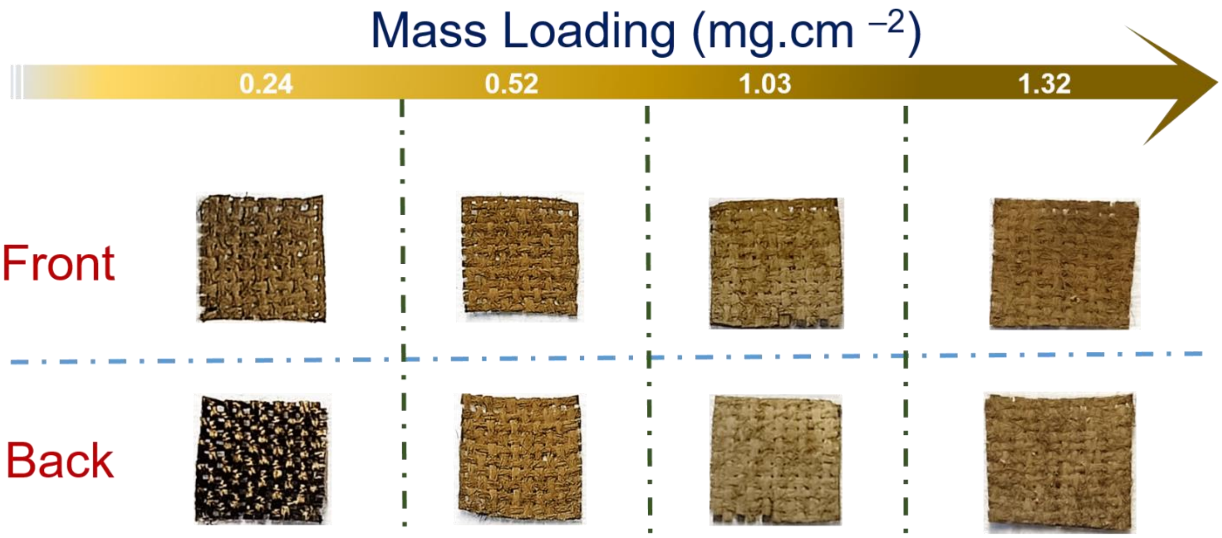

Figure S2. Digital images (back and front) of Si NWs@SSFC with different mass loadings. The mass loading of Si NWs@SSFc can be adjusted by using either a single or dual sided Sn-seed coated SSFC, reaction time and amount of PS.

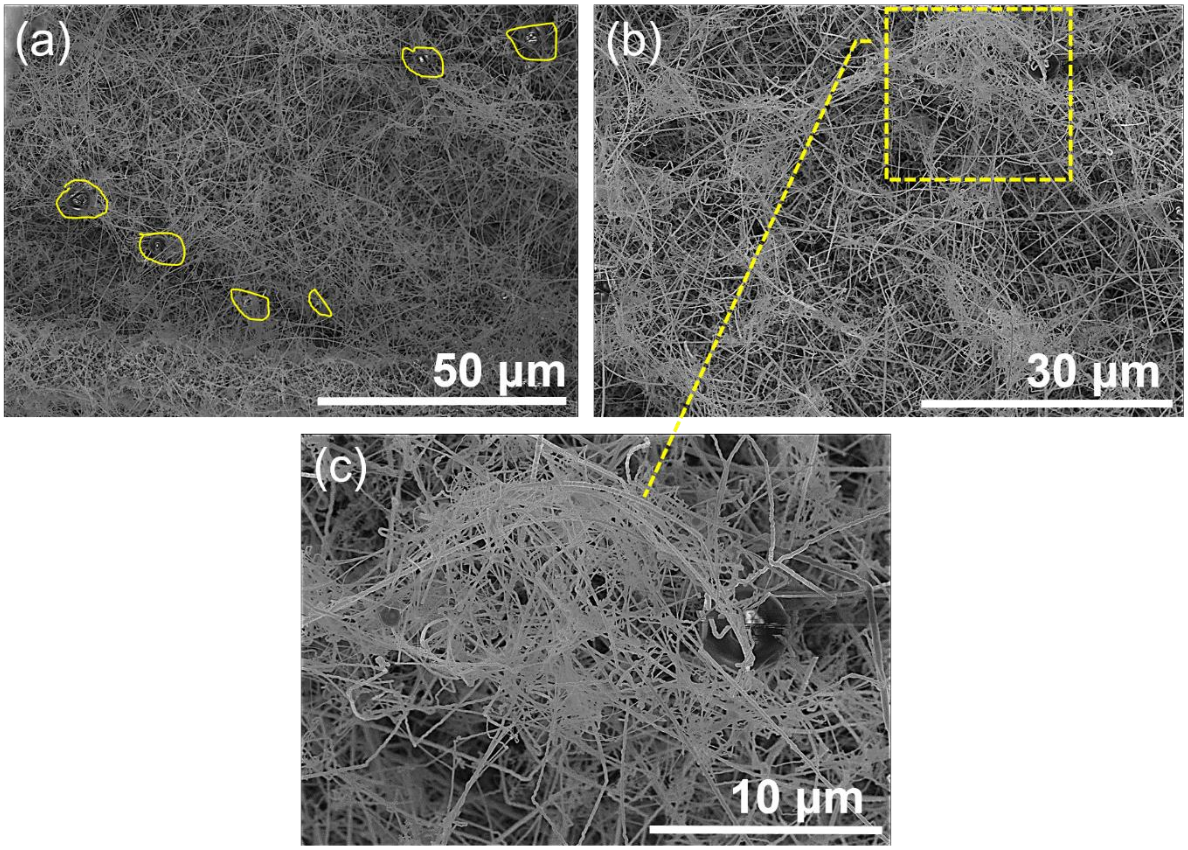

Figure S3. SEM image of 1.32 sample showing (a) the micrometre sized amorphous silicon particles, (b, c) clustering of the Si NWs.

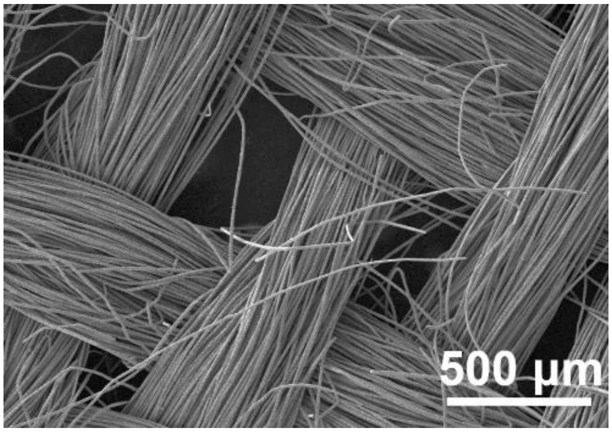

Figure S4. Low magnification SEM image of SSFC after the growth of Si NWs at 460 ^{o}C.

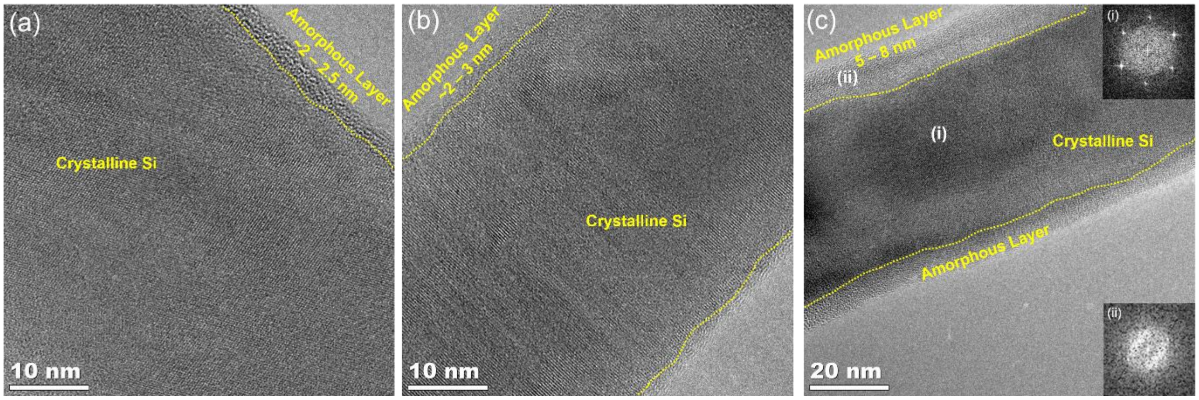

Figure S5. HRTEM images of Si NWs@SSFC with mass loading of (a) 0.52, (b) 1.03, (c) 1.32 mg.cm^{-2}, with the growth time of 10, 15, and 30 minutes, respectively.

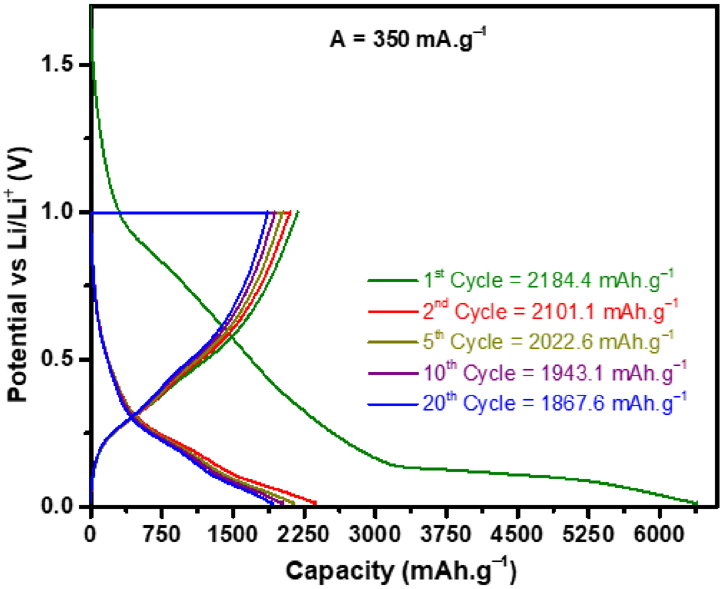

Figure S6. Determination of experimental specific capacity of Sn Seeded Si NWs@SSFC (0.23 mg cm^{-2}). Si NWs were cycled in the voltage window of 0.01–1.0V vs Li/Li^{+} at a constant current density of 350 mA.g^{-1}. Based on that an experiential specific capacity of 2200 mAh.g^{-1} was used as an appropriate capacity to calculate suitable C–rates for electrochemical testing, where C–rates C/10, C/5, C/2, 1C, 2C and 5C, corresponds to the current densities of 220, 440, 1100, 2200, 4400, and 11000 mA.g^{-1} respectively.

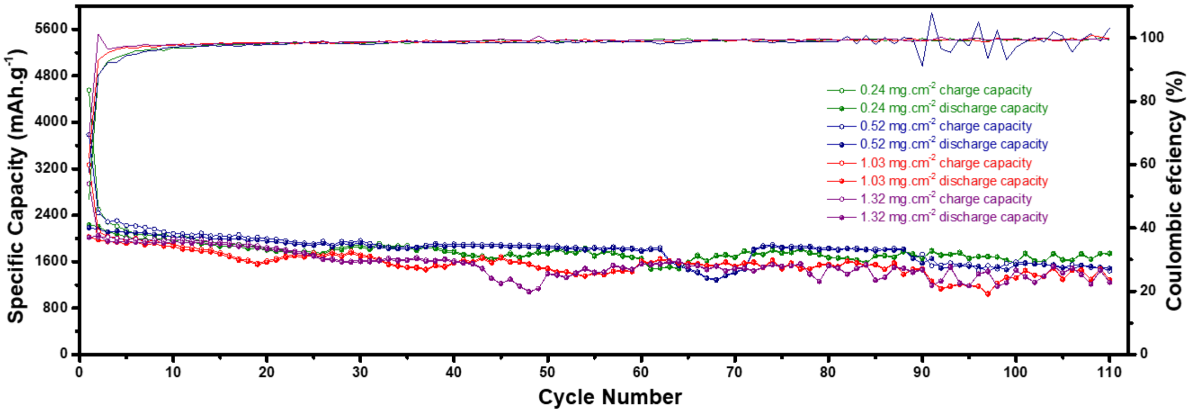

Figure S7. Cycling performance of 0.24, 0.52, 1.03 and 1.32 mg.cm^{-2} samples at C/5

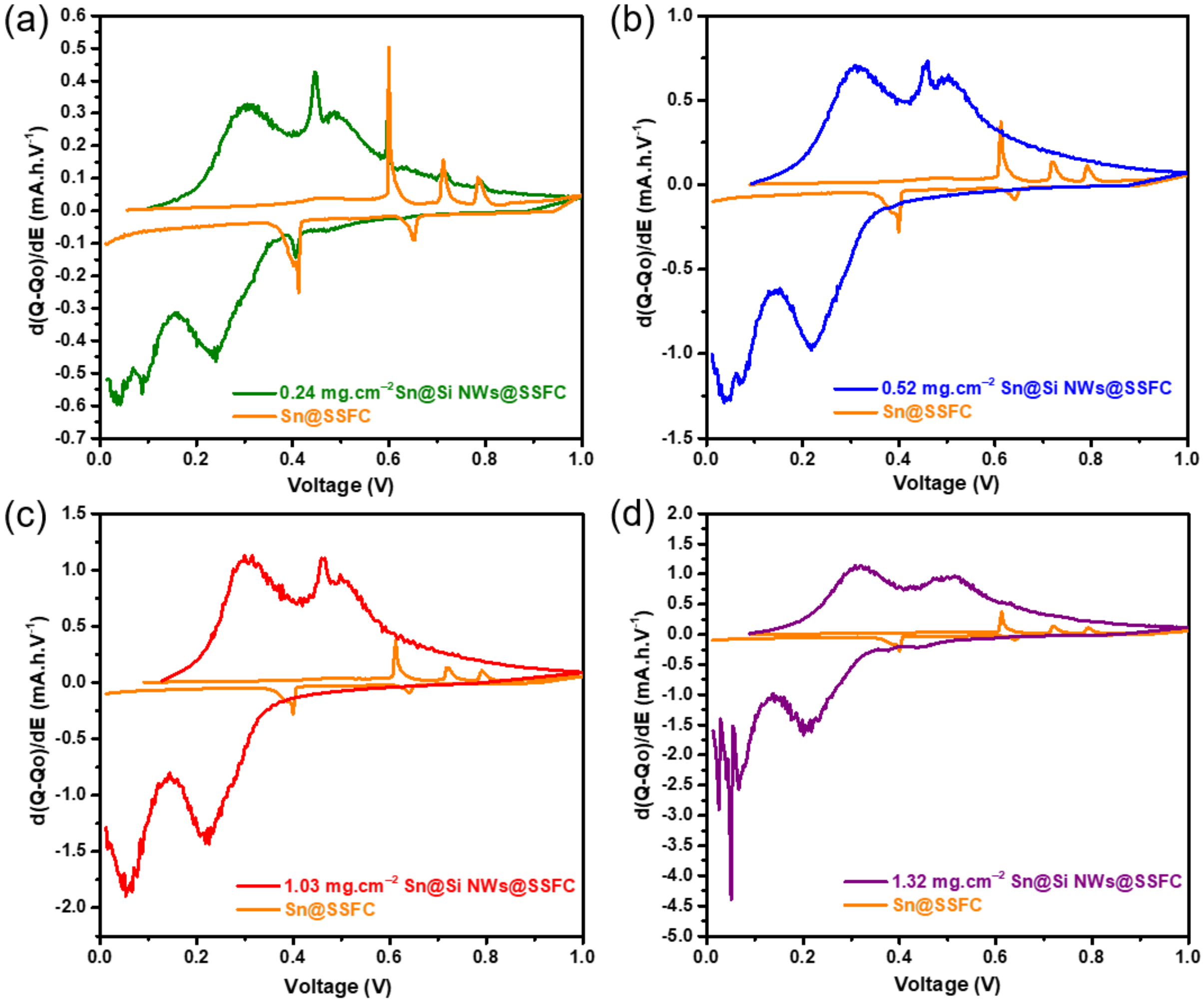

Figure S8. Differential capacity plot of the second cycle for the Sn-seeded Si NW@SSFC electrode for (a) 0.24, (b) 0.52, (c) 1.03, and (d) 1.32 mg.cm^{-2} samples. The orange line in all four is the Sn layer at SSFC electrode on its own.

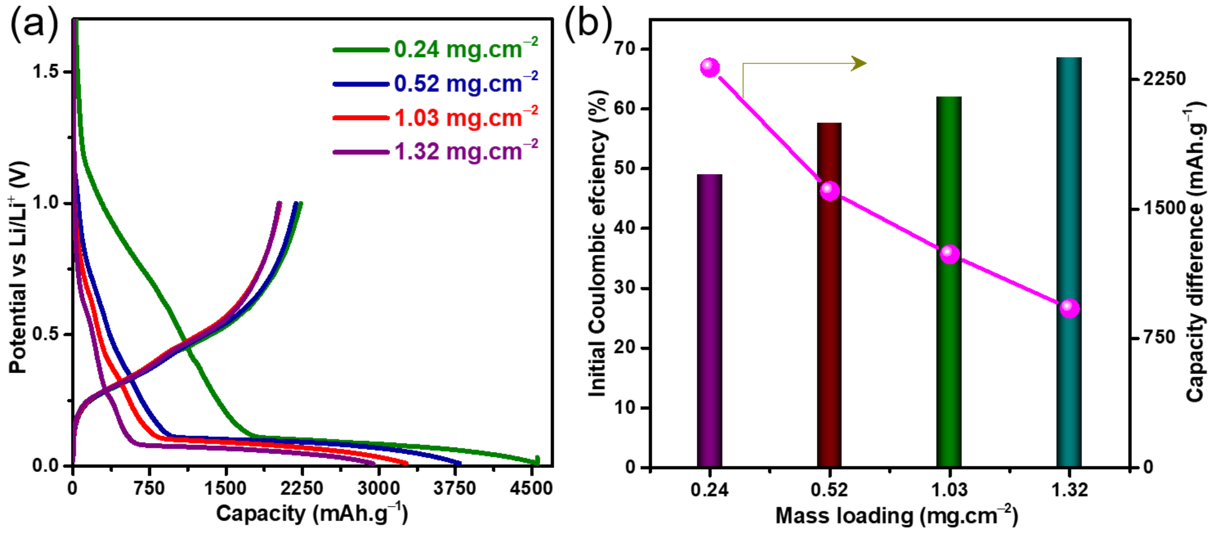

Figure S9. (a) Initial Galvanostatic charge/discharge voltage profiles for 0.24, 0.52, 1.03 and 1.32 mg.cm^{-2} at C/5, and (b) Comparison of the initial Coulombic efficiency and capacities different during first cycle.

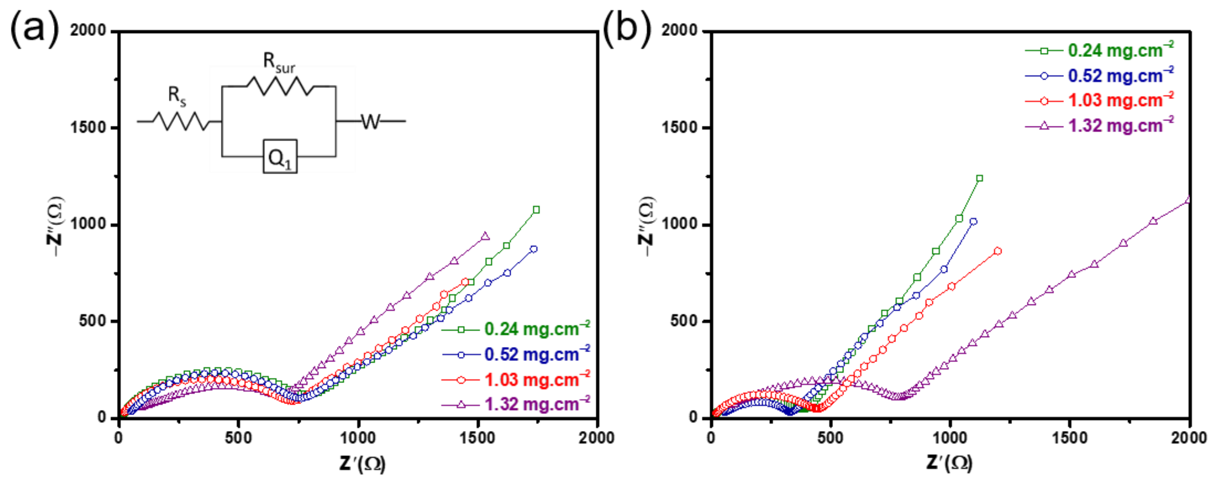

Figure S10. Comparison of EIS after (a) 1st cycle, and (b) 10th cycle for 0.24, 0.52, 1.03, and 1.32 mg.cm^{-2} electrode. The inset represents the equivalent circuit used in the analysis

Table S1. R_{sur} values for different loadings after 1st and 10th cycle

Mass Loading of Si NWs@SSFC	After 1 Cycle R_{sur} (Ω)	After 10 Cycles R_{sur} (Ω)
**0.24 mg.cm^{-2}**	746.9	367.1
**0.52 mg.cm^{-2}**	724.3	316.8
**1.03 mg.cm^{-2}**	693.1	421.5
**1.32 mg.cm^{-2}**	621.6	755.5

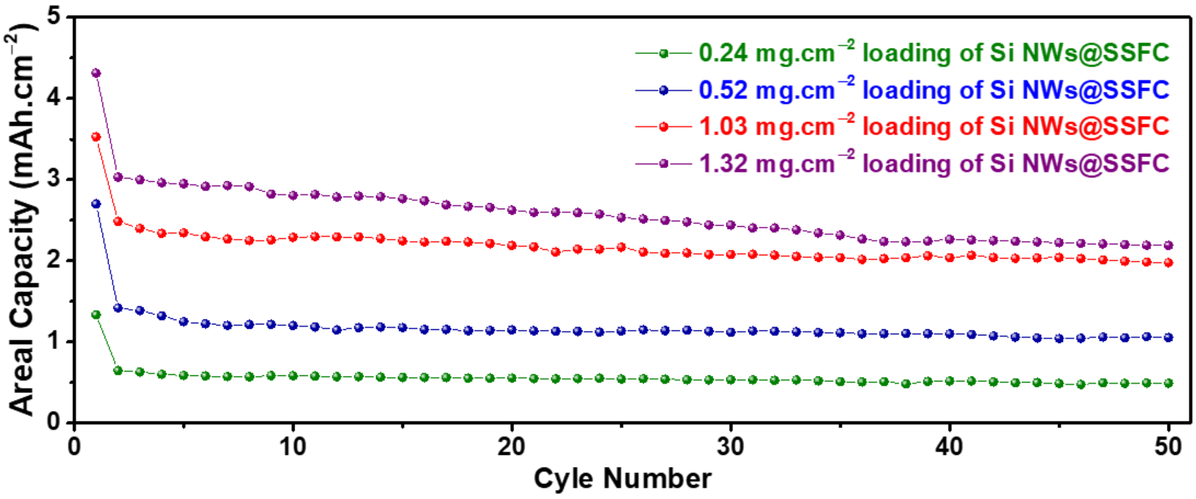

Figure S11. Areal capacity performance of the electrodes with various mass loadings at C/10

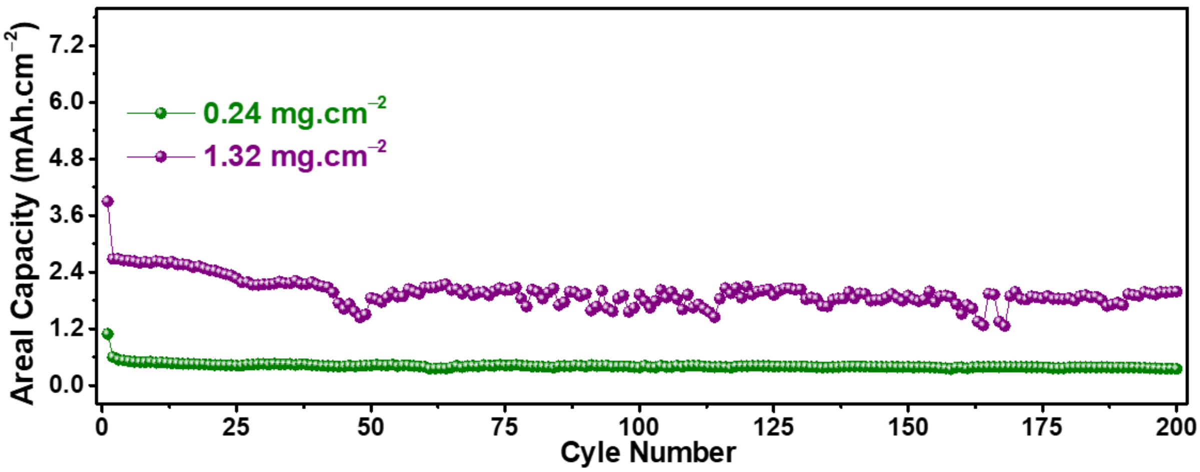

Figure S12. Areal capacity vs cycle number of Si NWs@SSFC with mass loadings 0.24 and 1.32 mg.cm^{-2} at C/5

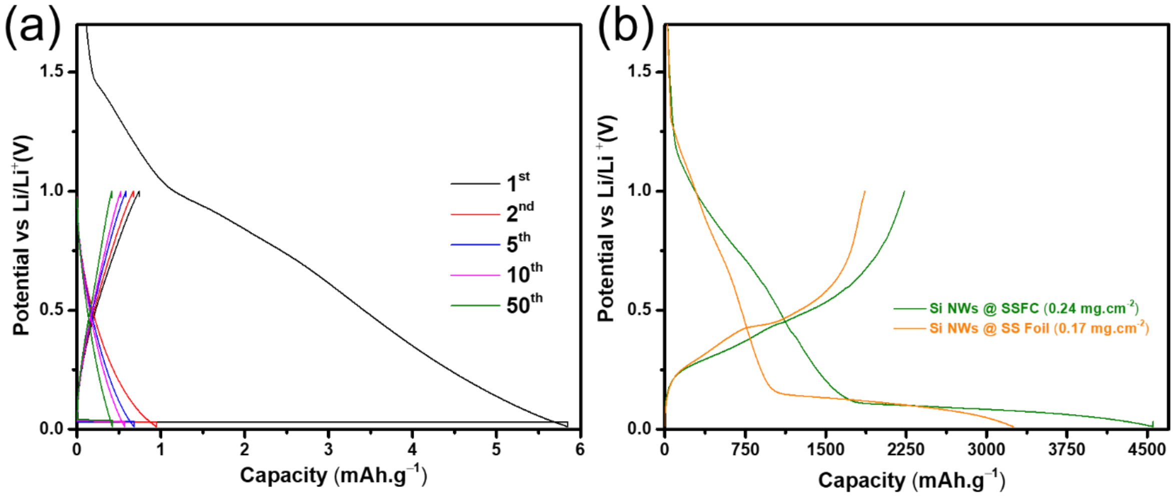

Figure S13. Galvanostatic charge/discharge profiles of (a) blank SSFC, and (b) Si NWs@SSFC vs Si NWs@SS foil with comparable loadings

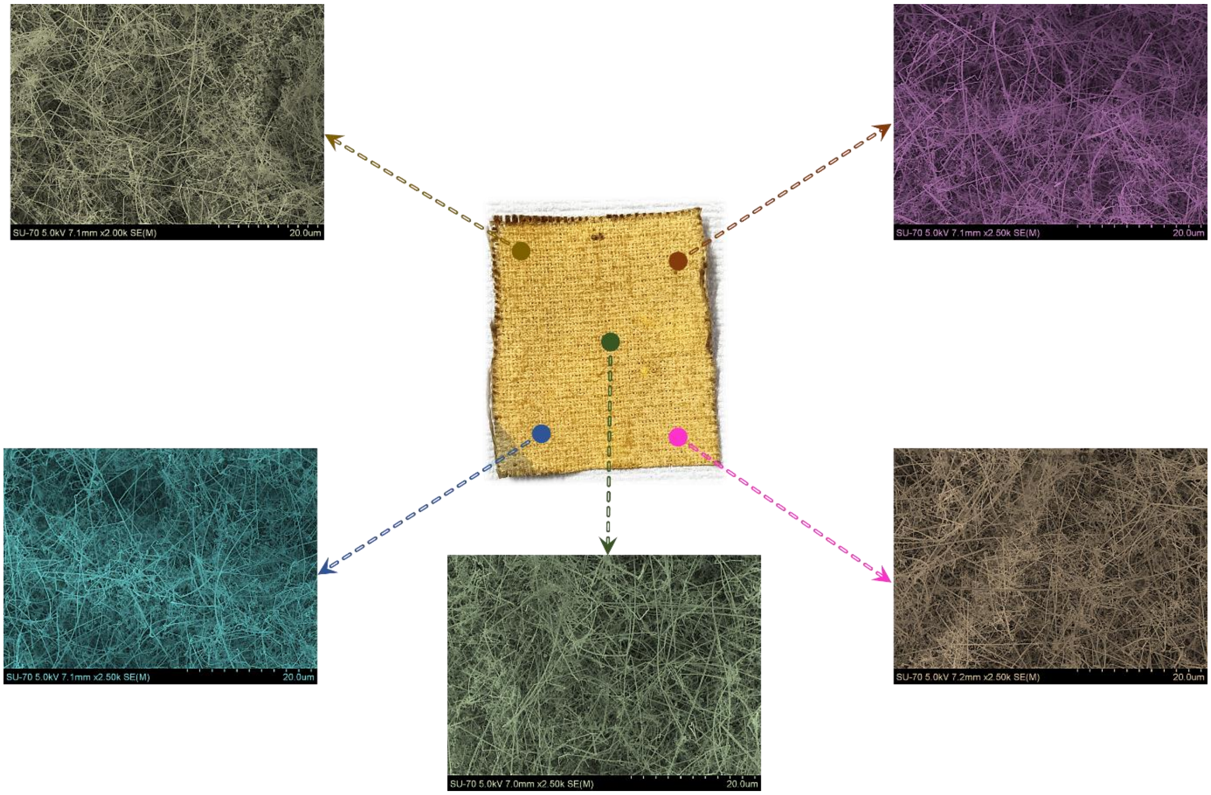

Figure S14. SEM image of the Si NWs@SSFC taken from the areas indicated by the dots in the photograph after intense mechanical folding, twisting, and rolling.

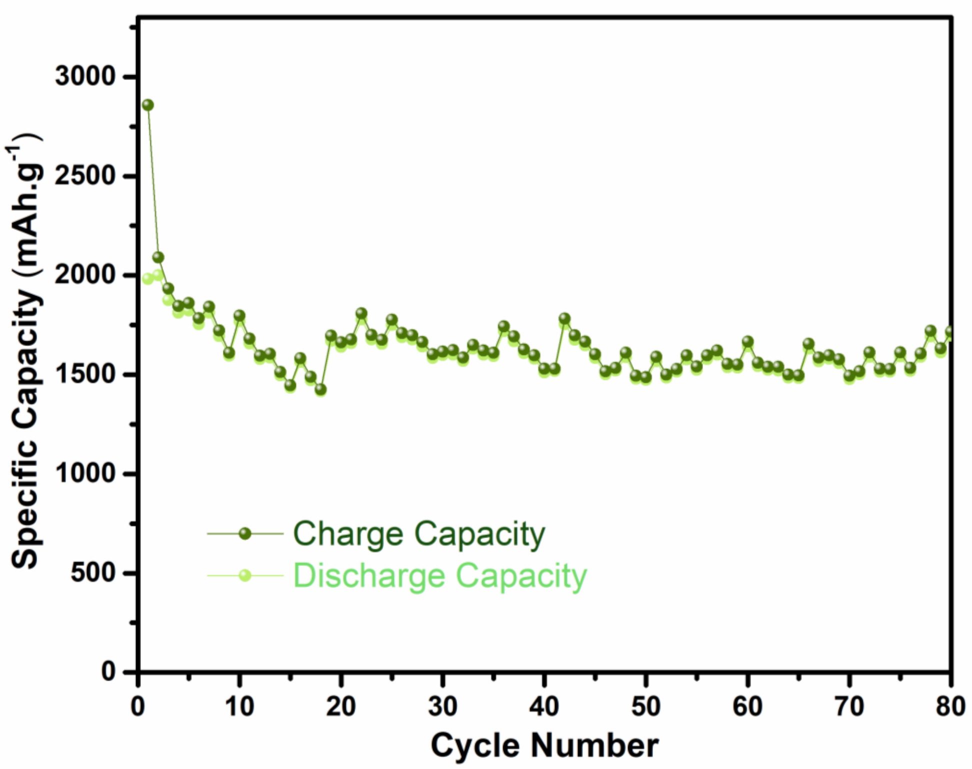

Figure S15. Cycling performance of Si NWs@SSFC after intense mechanical handling

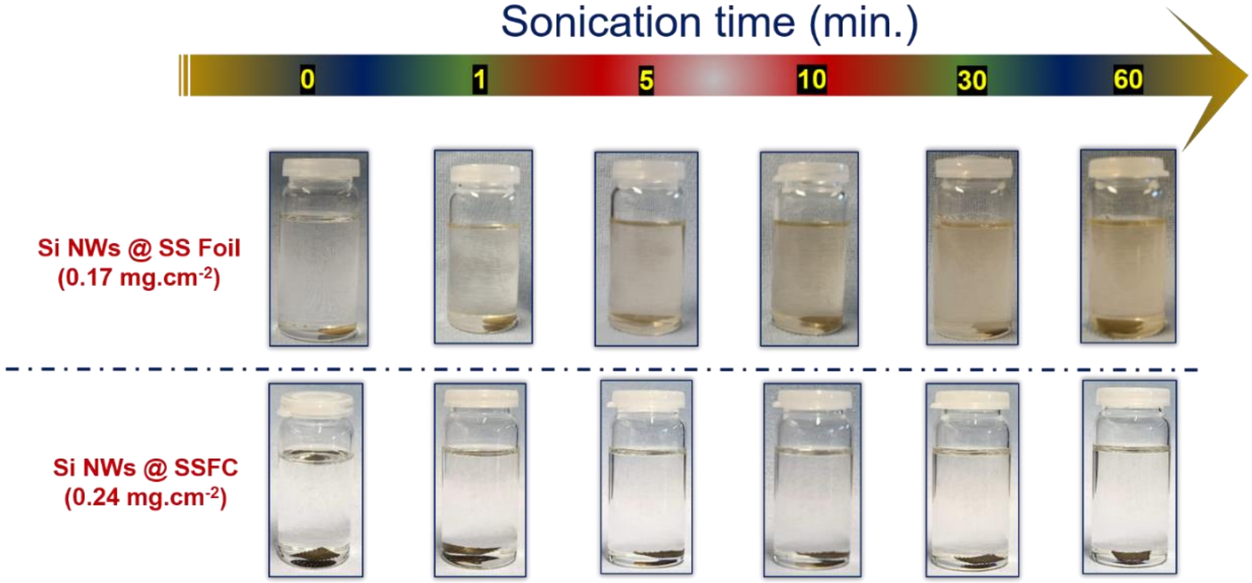

Figure S16. Photographs of Si NWs@SS foil and Si NWs@SSFC taken before and after 1, 5, 10, 30 and 60 minutes of sonication.

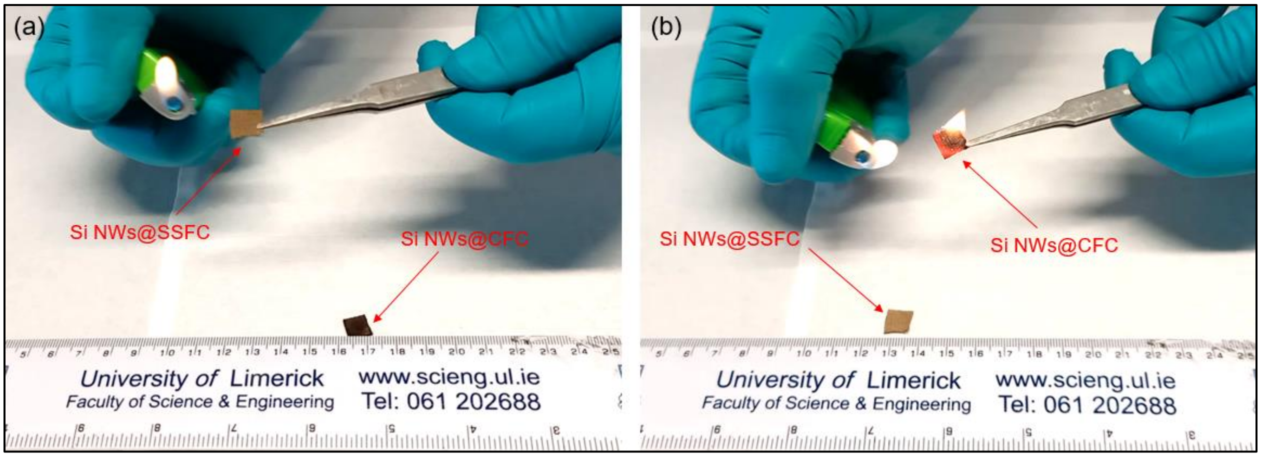

Figure S17. Flammability test for (a) Si NWs@SSFC, and (b) Si NWs@CC showing that SSFC is flame resistant (See Video S2 for more details)

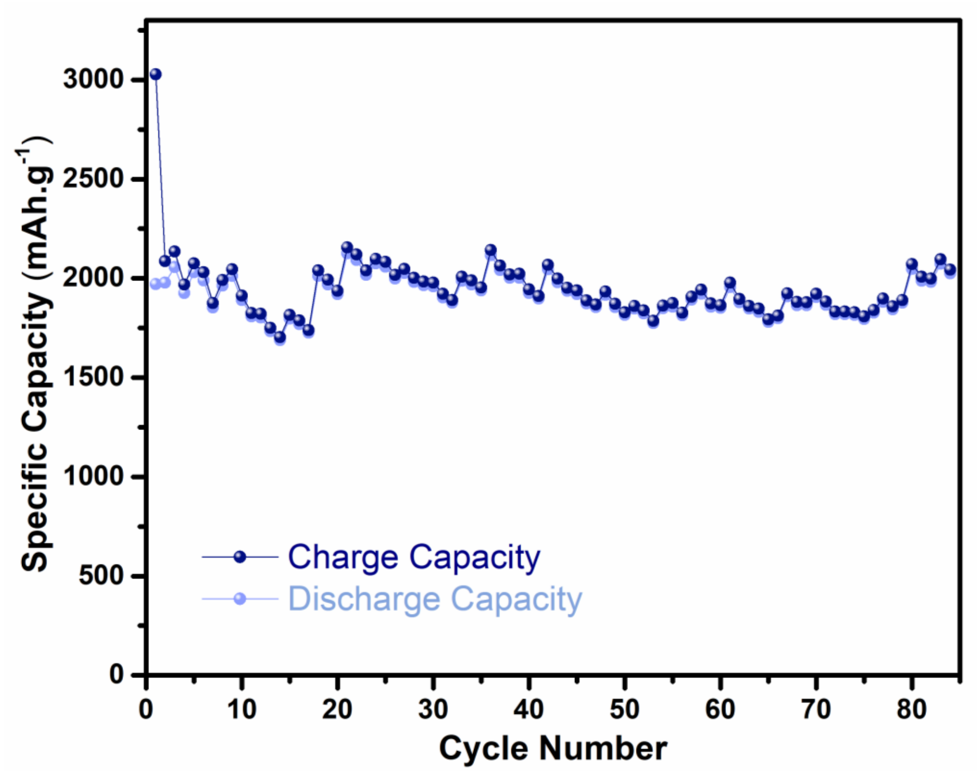

Figure S18. Cycling performance of Si NWs@SSFC anode after exposure to fire

(a)

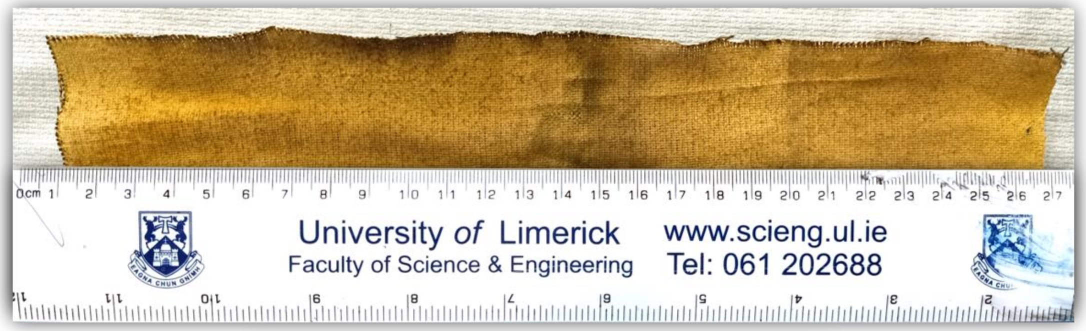

(b)

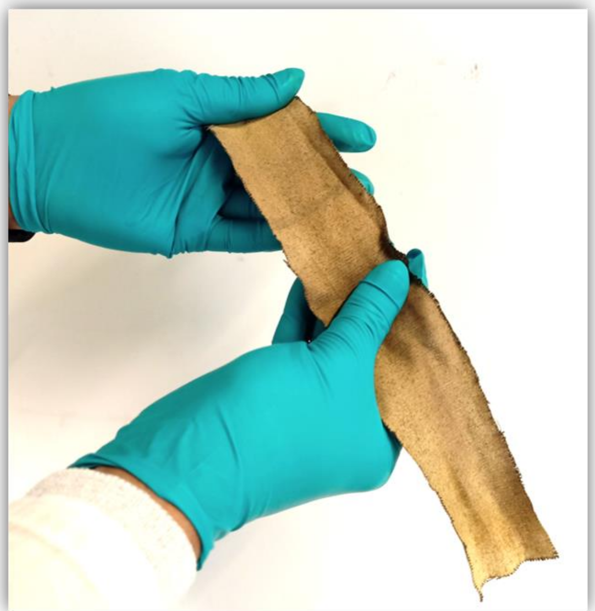

Figure S19. (a, b) photograph of large sized 3D Si NWs@SSFC anode (130 cm^2) prepared using 100 mL on long–necked round bottom flask

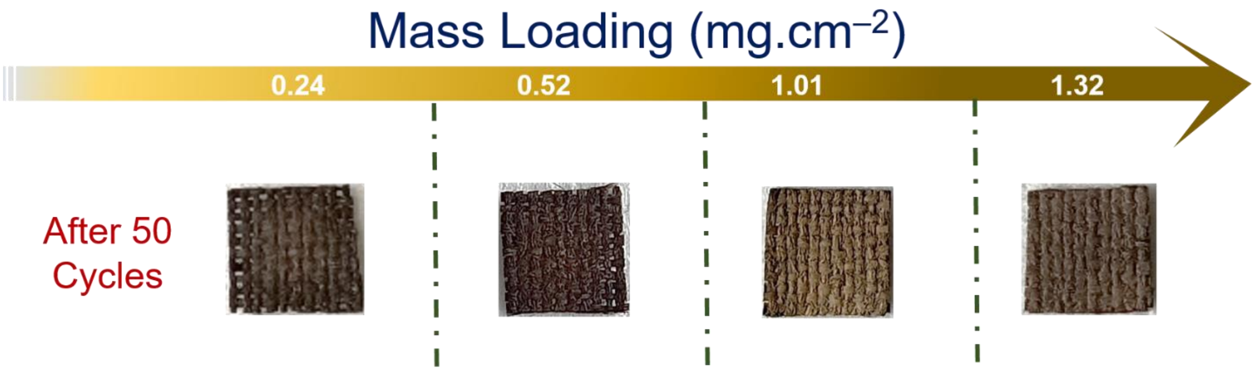

Figure S20. Digital images of Si NWs@SSFC anodes with different mass loadings after 50 cycles. The anodes were washed with acetonitrile after cycling and dried in oven before taking pictures. The change in the colour of anodes after cycling compared to the pristine samples as presented in Figure S2 possibly due to the electrochemical reactions comprising of electrolyte decomposition and/or oxidation.

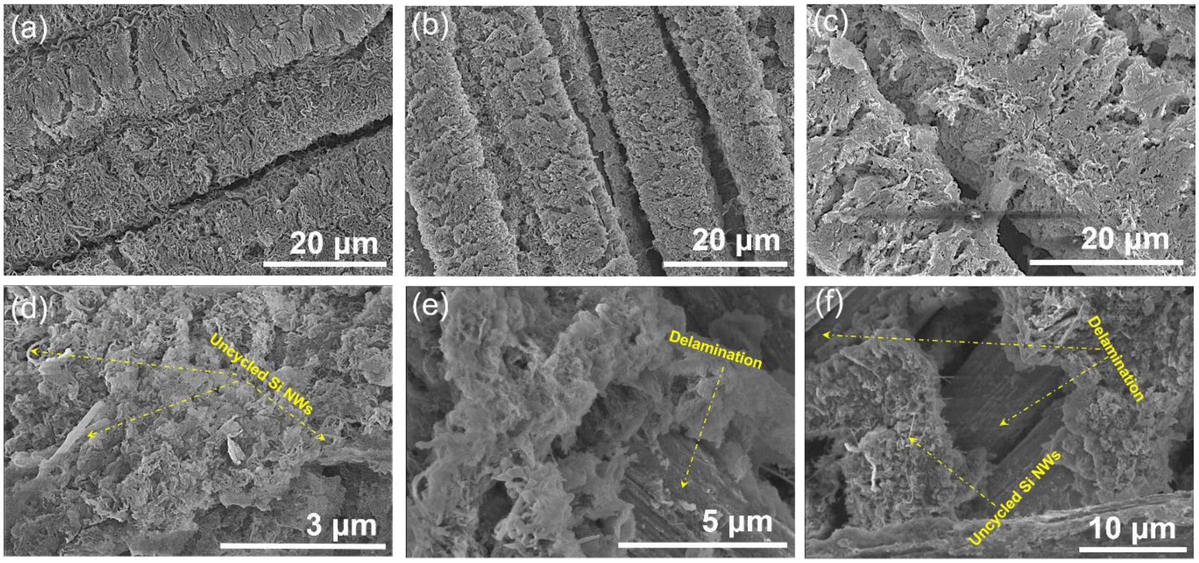

Figure S21. Low magnification *ex–situ* SEM images of (a) 0.24, (b) 0.52, (c) 1.03, and (d–f) 1.32 mg.cm^{-2} Si NWs@SSFC anodes after 50 cycles

Table S2. A Comparison of the mass loadings, initial Gravimetric and areal capacities, and retentive areal capacity for previously published Si based binder–free, composites and conventional slurry based anodes

Anode Material	Mass Loadings ($mg.cm^{-2}$)	Initial Gravimetric Capacity ($mAh.g^{-1}$)	Initial Areal Capacity ($mAh.cm^{-2}$)	Areal Capacity retention ($mAh.cm^{-2}$)	No. of Cycles	References
Si NWs@SSFC	1.32	2019	3.9	2.0	200	***This Work***
Si NWs-grown-on-graphite	2.7	~1650	~4.1	~2.0	100	***ACS nano*** 14, 12006 (**2020**).[S1]
Multilayer Si/CNT coaxial nanofiber	–	~3000	~1.9	1.25	50	***Energy Environ. Sci.*** 7, 655 (**2014**).[S2]
Si NWs@carbon	1.06	3100	3.7	~3	27	***Nano Lett.*** 15, 3907 (**2015**).[S3]
Si@Porous copper	0.10	910	0.75	–	60	***Solid State Ion.*** 288, 204 (**2016**).[S4]
Si NWs–CNT	0.5	2400	1.6	~0.9	35	***Adv. Energy Mater.*** 2, 87 (**2012**).[S5]
Si nanoparticle decorated Si NWs	~0.3	1600	~2.5	~1.85	50	***Chem. Commun.*** 47, 367 (**2011**).[S6]
Ant-nest-like porous silicon	2.0	~2843	~4.0	~2.5	100	***Nat. Commun.*** 10, 1447 (**2019**).[S7]
Carbon Bridged Oriented Graphene-Si Nanoparticles	1.90	1390	2.50	2.40	200	***Nano Lett.*** 15, 6222 (**2015**).[S8]
Si-based multicomponent	1.5	1000	–	1.5	1000	***Energy Environ. Sci.*** 8, 2075 (**2015**).[S9]
3D porous Si	0.231	2680	~0.62	0.42	70	***Nano Lett.*** 12, 6060 (**2012**).[S10]
Si nanoparticles layer	2.2	~900	~4	1.5	100	***Nano Energy*** 38, 477 (**2017**).[S11]
Si pomegranate @ void @ Carbon	2.34	2350	~2.7	~2.10	1000	***Nat. Nanotechnol.*** 9, 187 (**2014**).[S12]
Fe–Cu–Si ternary composite	8.5	1287	3.44	~2.0	300	***Energy Environ. Sci.*** 9, 1251 (**2016**).[S13]
Mesoporous Si sponge	0.5	~900	~3.5	~1.5	300	***Nat. Commun.*** 5, 4105 (**2014**).[S14]
Si-PEDOT:PSS-CNT	2	2209	2.2	1.8	100	***Adv. Energy Mater.*** 4, 1400207 (**2014**).[S15]

Ge@aSi NWs	0.41	2066	0.85	0.44	150	***ACS Appl. Mater. Interfaces*** 11, 19372 (**2019**).[S16]
Watermelon Inspired Si/C Microspheres	4.1	620	2.54	1.91	500	***Adv. Energy Mater.*** 7, 1601481 (**2017**).[S17]

Note: For better and reasonable comparison, the mass loading of active material and cycling number must be taken into account.

References

[S1] S. Karuppiah, C. Keller, P. Kumar, P. H. Jouneau, D. Aldakov, J. B. Ducros, G. Lapertot, P. Chenevier, C. Haon, *ACS nano* **2020**, 14, 12006.

[S2] Q. Xiao, Y. Fan, X. Wang, R. A. Susantyoko, Q. Zhang, *Energy Environ. Sci.* **2014**, 7, 655.

[S3] E. Peled, F. Patolsky, D. Golodnitsky, K. Freedman, G. Davidi, D. Schneier, *Nano Lett.* **2015**, 15, 3907.

[S4] F. Dogan, L. D. Sanjeewa, S.-J. Hwu, J. T. Vaughey, *Solid State Ion.* **2016**, 288, 204.

[S5] X. Li, J.-H. Cho, N. Li, Y. Zhang, D. Williams, S. A. Dayeh, S. T. Picraux, *Adv. Energy Mater.* **2012**, 2, 87.

[S6] L. Hu, H. Wu, S. S. Hong, L. Cui, J. R. McDonough, S. Bohy, Y. Cui, *Chem. Commun.* **2011**, 47, 367.

[S7] W. An, B. Gao, S. Mei, B. Xiang, J. Fu, L. Wang, Q. Zhang, P. K. Chu, K. Huo, *Nat. Commun.* **2019**, 10, 1447.

[S8] M. Zhou, X. Li, B. Wang, Y. Zhang, J. Ning, Z. Xiao, X. Zhang, Y. Chang, L. Zhi, *Nano Lett.* **2015**, 15, 6222.

[S9] J.-I. Lee, Y. Ko, M. Shin, H.-K. Song, N.-S. Choi, M. G. Kim, S. Park, *Energy Environ. Sci.* **2015**, 8, 2075.

[S10] S. R. Gowda, V. Pushparaj, S. Herle, G. Girishkumar, J. G. Gordon, H. Gullapalli, X. Zhan, P. M. Ajayan, A. L. Reddy, *Nano Lett.* **2012**, 12, 6060.

[S11] Y. Xu, E. Swaans, S. Chen, S. Basak, P. P. R. M. L. Harks, B. Peng, H. W. Zandbergen, D. M. Borsa, F. M. Mulder, *Nano Energy* **2017**, 38, 477.

[S12] N. Liu, Z. Lu, J. Zhao, M. T. McDowell, H. W. Lee, W. Zhao, Y. Cui, *Nat. Nanotechnol.* **2014**, 9, 187.

[S13] S. Chae, M. Ko, S. Park, N. Kim, J. Ma, J. Cho, *Energy Environ. Sci.* **2016**, 9, 1251.

[S14] X. Li, M. Gu, S. Hu, R. Kennard, P. Yan, X. Chen, C. Wang, M. J. Sailor, J. G. Zhang, J. Liu, *Nat. Commun.* **2014**, 5, 4105.

[S15] Z. Chen, J. W. F. To, C. Wang, Z. Lu, N. Liu, A. Chortos, L. Pan, F. Wei, Y. Cui, Z. Bao, *Adv. Energy Mater.* **2014**, 4, 1400207.

[S16] K. Stokes, W. Boonen, H. Geaney, T. Kennedy, D. Borsa, K. M. Ryan, *ACS Appl. Mater. Interfaces* **2019**, 11, 19372.

[S17] Q. Xu, J.-Y. Li, J.-K. Sun, Y.-X. Yin, L.-J. Wan, Y.-G. Guo, *Adv. Energy Mater.* **2017**, 7, 1601481.
